# Molecular Mechanisms in Pentanucleotide Repeat Diseases

**DOI:** 10.3390/cells11020205

**Published:** 2022-01-08

**Authors:** Joana R. Loureiro, Ana F. Castro, Ana S. Figueiredo, Isabel Silveira

**Affiliations:** 1 Genetics of Cognitive Dysfunction Laboratory, i3S- Instituto de Investigação e Inovação em Saúde, Universidade do Porto, 4200-135 Porto, Portugal; joana.loureiro@ibmc.up.pt (J.R.L.); afcastro@i3s.up.pt (A.F.C.); asfigueiredo@ibmc.up.pt (A.S.F.); 2 Institute for Molecular and Cell Biology, Universidade do Porto, 4200-135 Porto, Portugal; 3 Instituto de Ciências Biomédicas Abel Salazar, Universidade do Porto, 4050-313 Porto, Portugal

**Keywords:** ATTTC repeat insertion, spinocerebellar ataxia, SCA37, familial adult myoclonic epilepsy, FAME1, polyglutamine, polyalanine, RNA-binding protein, RNA foci, liquid–liquid phase separation

## Abstract

The number of neurodegenerative diseases resulting from repeat expansion has increased extraordinarily in recent years. In several of these pathologies, the repeat can be transcribed in RNA from both DNA strands producing, at least, one toxic RNA repeat that causes neurodegeneration by a complex mechanism. Recently, seven diseases have been found caused by a novel intronic pentanucleotide repeat in distinct genes encoding proteins highly expressed in the cerebellum. These disorders are clinically heterogeneous being characterized by impaired motor function, resulting from ataxia or epilepsy. The role that apparently normal proteins from these mutant genes play in these pathologies is not known. However, recent advances in previously known spinocerebellar ataxias originated by abnormal non-coding pentanucleotide repeats point to a gain of a toxic function by the pathogenic repeat-containing RNA that abnormally forms nuclear foci with RNA-binding proteins. In cells, RNA foci have been shown to be formed by phase separation. Moreover, the field of repeat expansions has lately achieved an extraordinary progress with the discovery that RNA repeats, polyglutamine, and polyalanine proteins are crucial for the formation of nuclear membraneless organelles by phase separation, which is perturbed when they are expanded. This review will cover the amazing advances on repeat diseases.

## 1. Introduction

The number of hereditary disorders originated by unstable microsatellite repeat tracts has been rapidly increasing. Most of them are neurological, neuromuscular, or neurodegenerative diseases. Recent work has shown that a growing group of these conditions is caused by global RNA dysregulation [1,2,3]. The identified RNA alterations modify transcription, generation of antisense RNA, RNA processing, RNA translation, and RNA nuclear export [4,5,6,7]. The fact that most of the genes harboring repeat tracts have a role in transcription or are transcription factors (TFs) also points for a crucial role of RNA processing dysregulation in repeat diseases.

**Figure 1:**
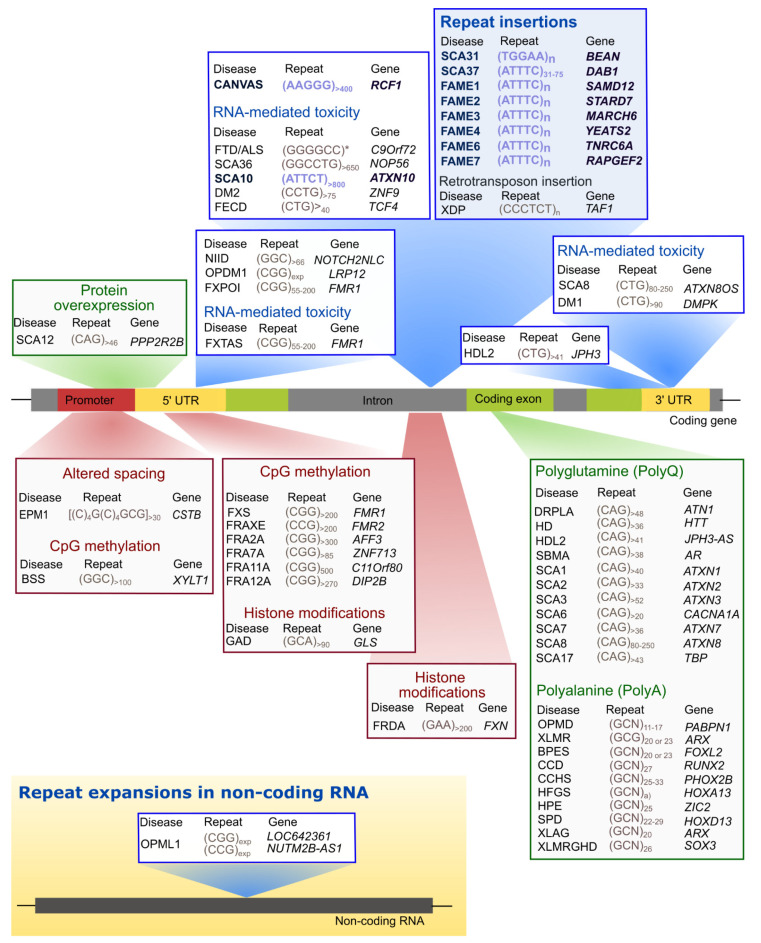
Genomic localization of pathogenic repeat expansions. Pathogenic repeats are found in protein-coding genes or non-coding RNAs; in gene-coding regions, these repeats are found in promoters, 5′ UTRs, exons, introns and 3′ UTRs; in red boxes are represented expanded repeats that trigger gene silencing or downregulation (gene loss-of-function); in blue boxes the non-coding transcribed repeat expansions (RNA-mediated toxicity); in green boxes are expanded repeats in protein coding regions or that lead to upregulation (protein gain-of-function). The pentanucleotide repeats are highlighted in blue. In promoters are located the pathogenic repeats causing progressive myoclonus epilepsy of the Unverricht-Lundborg (EPM1) [8] and Baratela-Scott Syndrome (BSS) [9]; the repeat expansion leading to spinocerebellar ataxia type 12 (SCA12) is in the promoter or the 5’ UTR, depending on the transcript [10,11]. In 5′ UTRs are found the expanded repeats associated with fragile X syndrome (FXS or FRAXA) [12], FRAXE [13], FRA2A [14], FRA7A [15], FRA11A [16], FRA12A [17], glutaminase deficiency (GAD) [18], fragile-X-associated tremor ataxia syndrome (FXTAS) [19], fragile X-associated primary ovarian insufficiency (FXPOI) [20], neuronal intranuclear inclusion disease (NIID) [21,22,23], and oculopharyngodistal myopathy (OPDM1) [23]. In introns, abnormal repeat tracts may lead to Friedrich ataxia (FRDA) [24], frontotemporal dementia/amyotrophic lateral sclerosis (FTD/ALS) [25,26], SCA10 [27], SCA36 [28,29], myotonic dystrophy type 2 (DM2) [30], and Fuchs endothelial corneal dystrophy (FECD) [31]. The pathogenic pentanucleotide repeats causing cerebellar ataxia with neuropathy and vestibular areflexia (CANVAS) [32,33], SCA31 [34], SCA37 [35], familial adult myoclonic epilepsy type 1 (FAME1) [36], FAME2 [37], FAME3 [38], FAME4 [39], FAME6, and FAME7 [36] are also intronic; a repeat-containing retrotransposon insertion potentially interferes with transcription of surrounding genomic elements in X-linked dystonia-parkinsonism (XDP) [40]. In 3′ UTR, expanded trinucleotide repeats originate DM1 [41,42]. The Huntington’s disease-like 2 (HDL2) repeat expansion is located both in the 3′ UTR and in an exon, depending on the transcript, being bidirectional transcribed [43,44,45] as in SCA8 [46,47]. In exons, trinucleotide repeat expansions encode abnormal polyglutamine (polyQ) tracts in dentatorubral-pallidoluysian atrophy (DRPLA) [48], HD [49], SCA1 [50], spinobulbar muscular atrophy (SBMA) [51], SCA2 [52], Machado-Joseph disease/SCA3 [53], SCA6 [54], SCA7 [55], and SCA17 [56] or polyalanine (polyA) stretches in oculopharyngeal muscular dystrophy (OPMD) [57], X-linked mental retardation (XLMR) [58], blepharophimosis syndrome (BPES) [59], cleidocranial dysplasia (CCD) [60], congenital central hypoventilation syndrome (CCHS) [61], hand-foot-genital syndrome (HFGS) [62], holoprosencephaly (HPE) [63], synpolydactyly (SPD) [64], X-linked mental retardation and abnormal genitalia (XLAG) [65], or XLMR and growth hormone deficit (XLMRGHD) [66]. An expanded repeat in opposite DNA strands of non-coding RNAs *LOC642361*/ *NUTM2B-AS1* cause oculopharyngeal myopathy with leukoencephalopathy (OPML1) [23]. * The pathogenic repeat threshold is not clear in FTD/ALS, some studies consider >30 repeats.

These repeat tracts can be located in coding or non-coding gene regions. In protein-coding gene regions, only trinucleotide repeat expansions have been found, whereas within introns, promoters, 3’ and 5’ UTRs not only trinucleotide but also tetra-, penta-, hexa-, and dodecanucleotide repeats expanded above a given repeat size threshold have been identified in pathological chromosomes (Figure 1).

**Figure 2:**
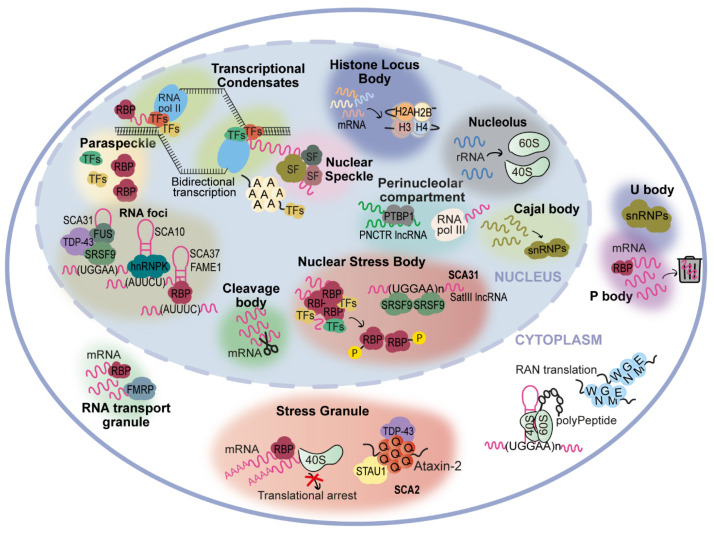
Representation of cellular condensates and location of RNA and proteins with expansions. Transcriptional condensates, formed by liquid–liquid phase separation, concentrate TFs, mediators, and other crucial players in transcription initiation. They are closely located to nuclear speckles, small reservoirs of splicing factors acting in mRNA splicing, and to paraspeckles, that assemble TFs and RBPs, thus regulating gene expression. Histone loci bodies drive histone synthesis and storage [71], while RNA transport granules carry mRNAs from and to the nucleus. The nucleolus is the place of rRNA synthesis and early-stage ribosomal subunit assembly. Under stress conditions (1) in nuclear stress bodies, the phosphorylation of RBPs leads to alterations in mRNA splicing of their target genes; in SCA31, the toxic (UGGAA)_n_ competes with SatIII (UGGAA)_n_ non-coding RNA for SRSF9 binding; (2) in the cytoplasm, the stress granules sequester mRNA, arresting translational initiation; Ataxin-2 protein has been implicated in the assembly of stress granules when expanded interacts with TDP-43 and recruits Staufen1 (STAU1) protein, leading to alterations in stress granule dynamics [4]. Perinucleolar compartment is thought to be the place where RNA Pol III transcripts are processed; additionally, recent work has demonstrated that the recruitment of PTBP1 by PNCTR long non-coding RNA controls the splicing patterns of RNA Pol II transcripts [72]; cleavage bodies are regions of mRNA processing. P bodies stall untranslated mRNAs, mediating its decay. U bodies assemble and store small nuclear ribonucleoproteins (snRNPs), previously biosynthesized in Cajal bodies. There are several mechanisms contributing to pathology in repeat expansion diseases (e.g., bidirectional transcription, RNA foci formation and RAN translation; most of them disturb phase-separation capacity, interfering with the correct assembly of condensates. Polyalanine—polyA; polyglutamine—polyQ.

There is evidence that expanded repeats in protein-coding gene regions could cause pathology by gain-of-function of mislocalized and aggregated polyglutamine [67] or polyalanine [68,69] proteins, many of them with both transcriptional and DNA repair functions, which could lead to their loss-of-function with disruption of DNA damage and repair processes [67,70] and/or interfere with the formation of condensates [3]. The abnormal DNA repair could explain the unstable nature of expanded repeat sequences that are characterized by changing their repeat size in parent-offspring transmissions and within a subject among tissues. The polyglutamine and polyalanine tracts in several proteins are in intrinsically disordered regions (IDR) involved in the formation of cellular membraneless condensates through a mechanism named liquid-liquid phase separation [3] (Figure 2). This is a spontaneous thermodynamically triggered mechanism in which two simultaneously existing liquid phases separate from one homogeneous solution. Several polyglutamine and polyalanine proteins have been demonstrated to form cellular condensates, by liquid-liquid phase separation, which have their composition and/or dynamics altered when these proteins are expanded [1,3,4]. Polyglutamine protein TBP binds to the TATA-box of gene promoters to trigger transcription, but when the polyglutamine tract is expanded, in spinocerebellar ataxia type 17 (SCA17), changes its phase separation capacity and probably its ability to co-condense with transcription co-activators (Figure 2), leading to transcriptional dysregulation, a mechanism implicated in many polyglutamine diseases [1,3,67,70]. Remarkably, Ataxin-2, an RNA-binding protein (RBP) harboring a polyglutamine sequence that when is expanded causes SCA2, has been implicated in the assembly of P-bodies and stress granules (Figure 2), two important cytoplasmic membraneless condensates involved in RNA processing [4]. Stress granule dynamics are perturbed by abnormal interaction of expanded Ataxin-2 with other proteins, which consequently aggregates [1,4]. Interestingly, in cytoplasmic aggregates, Ataxin-2 intermediate-length polyglutamine has been shown to interact with TDP-43, a nuclear RBP mislocalized in the cytoplasm in amyotrophic lateral sclerosis and other neurodegenerative diseases [1,5]. 

In non-coding gene regions, repeat expansions can originate epigenetic changes with transcriptional repression and protein loss-of-function such as in fragile X syndrome [73], due to CpG methylation of the (CGG)n, and Friedreich ataxia [74], caused by histone hypermethylation of the *FXN*. In Unverricht-Lundborg progressive myoclonus epilepsy (EPM1), a repeat expansion impairs transcription by altering the spacing of promoter elements. On the other hand, transcribed repeat expansions from non-coding gene sequences cause disease by a complex mechanism involving a toxic RNA gain-of-function through the production of an expanded RNA repeat that leads to the formation of nuclear RNA foci recruiting RBPs and originating splicing misregulation [7,75,76]. In cells, RNA foci have been shown to be formed by phase separation of the repeat-containing RNA [77]. RNAs transcribed from many repeat expansion loci, namely with AT-rich repeat motifs, have common properties of being able to form an RNA hairpin and due to their nucleotide repetitive nature drive multivalent intermolecular interactions [1,77]. The multivalent base-pairing causes expanded RNA repeats to undergo an abrupt sol–gel transition in vitro [77]. In cells, repeat-containing RNAs form aberrant nuclear RNA foci that display liquid-like properties [77]. The splicing misregulation is originated by loss-of-function of aberrantly recruited RBPs, for the RNA foci, most of them are transcription or splicing factors, resulting in the production of abnormal protein isoforms crucial for biological processes in the affected tissue [1], being able to interfere with phase separation capacity, as they have other amino acids with different physical or chemical properties [72]. These expanded non-coding RNA repeats abnormally recruit RBPs with a role in normal physiology [2], disturbing the biological function of these RNAs in the guidance of RBPs for the formation of nuclear compartments [71], consequently impacting genome regulation [78] and possibly the instability in non-coding repeat diseases.

The high complexity of mechanisms involved in repeat diseases is demonstrated by the fact that an expanded RNA repeat transcribed from both protein-coding and non-coding gene regions can lead to the formation of toxic peptides by repeat-associated non-AUG initiated (RAN) translation [6,79]. These RAN peptides have already been found in affected tissue of tri-, tetra-, penta-, and hexanucleotide repeat diseases [6].

To further increase the complexity of repeat expansion loci, they can be transcribed in sense and antisense orientations, originating neurotoxic sense and antisense expanded RNA repeats that, in addition, can be translated by RAN in all reading frames, producing toxic peptides [47,79,80,81,82,83]. The first antisense RNA repeat has been found in myotonic dystrophy type 1 (DM1) [81] followed, soon, by spinocerebellar ataxia type 8 (SCA8) [47], but currently, they are known for at least 14 diseases [84].

Interestingly, many repeat expansion pathologies are caused by expansions that have arisen in genes with TF activity as can be observed in Figure 1. Remarkably, TFs, transcriptional co-activators, and several RBPs composing the transcriptional machinery have recently been shown to have IDRs, leading to the formation of transcriptional condensates important for the recruitment of RNA polymerase II [85]. The presence of repeat expansions in TF IDRs may alter their phase separation capacity, which is necessary for the correct assembly of these transcriptional condensates. Disruption of the phase separation capacity of TFs encoded by TF genes involved in repeat expansion diseases has recently been demonstrated by Basu and colleagues for *HOXA13*, *HOXD13*, *RUNX2*, and *TBP* [3]. This evidence, combined with the high number of TFs with expanded repeats, suggests that the disruption of transcriptional condensates could be a common mechanism underlying many repeat expansion diseases.

**Figure 3:**
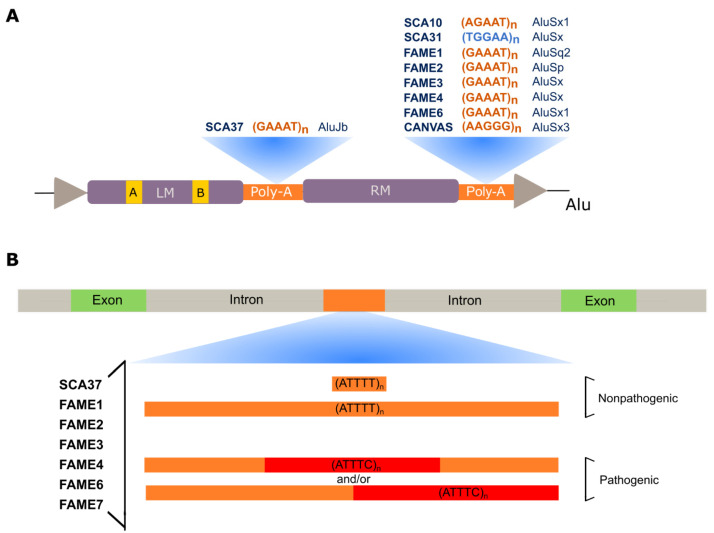
Pathogenic pentanucleotide repeats. (**A**) Schematic representation of pathogenic pentanucleotide repeats located in poly-A regions of ancient Alu families. Repeats are represented in Alu orientation; orange for repeats in genes with the Alu in antisense DNA strand; blue for repeats with Alu in shared introns of genes transcribed in opposite directions. The SCA37 repeat is located in the middle poly-A of an AluJb and the remaining are in 3′ poly-A tails of AluS elements. LM—left Alu monomer; RM—right Alu monomer; boxes A and B—internal regions of an RNA pol III promoter degenerated in ancient Alu families. (**B**) Schematic representation of non-pathogenic and pathogenic alleles with ATTTC repeat insertions, in intronic gene regions. Orange represents repeat composition of non-pathogenic alleles, whereas red is the pathogenic repeat insertion. Most non-pathogenic chromosomes have less than 30 repeats, but some can be larger than the pathogenic tract.

During the last decade, a new type of repeat expansion disease has emerged (Figure 3). These diseases have arisen due to a nucleotide substitution in a non-pathogenic pentanucleotide repeat tract, originating a novel and pathogenic pentanucleotide repeat insertion with adjacent or flanking non-pathogenic pentanucleotide repeats [34,35,86,87]. This type of repeat insertion was first identified in SCA31, in 2009 [34]. Later, in 2017, the second repeat insertion of a new pentanucleotide repeat motif was found causing SCA37 [35]. More recently, ATTTC pentanucleotide repeat insertions have been discovered in six genes causing familial adult myoclonic epilepsies (FAME) type 1, 2, 3, 4, 6, and 7 [36,37,38,39].

This review focuses on pentanucleotide repeat insertions and their contribution to the current understanding of how repeat expansions cause neurodegenerative diseases.

## 2. Pentanucleotide Repeats in Spinocerebellar Ataxias

The SCAs are a genetically heterogeneous group of usually adult onset autosomal-dominant neurodegenerative diseases characterized by progressive loss of balance and coordination, accompanied by slurred speech. The most common SCAs are caused by repeat expansions in coding or non-coding gene regions, but an unknown proportion of these disorders result from non-repeat mutations. Both groups of SCAs have already been reviewed [84,88,89,90], we will cover the SCAs originated by pentanucleotide repeat inserted mutations.

### 2.1. ATXN10 ATTCT Repeat Expansions and Inserted Interruptions in SCA10

SCA10 is characterized by cerebellar atrophy, ataxia, and seizures [91]. This disease is one of the most common SCAs in Mexico and Brazil [27,92,93,94,95] and has been reported in diverse North, Central, and South American populations [96,97,98,99] and Asia [100,101]. SCA10 is caused by an (ATTCT)_n_ expansio_n_ in intron 9 of *ATXN10* [27], a gene required for the survival of cerebellar neurons inducing neuritogenesis [102]. Non-pathogenic alleles range from 10 to 32 repeats, whereas expanded alleles in affected individuals vary from 800 to 4500 repeats (Table 1). Intermediate-size alleles with reduced penetrance have also been reported [93,99,103]. Remarkably, in subjects with epileptic seizures, the repeat expansion is interrupted by inserted pentanucleotide motifs such as (ATTCC)_n_, (ATCCC)_n_, and (ATCCT)_n_ that modify disease presentation [103,104,105,106]. Interestingly, the pathogenic repeat is in the poly-A tail of an Alu DNA element [107] similar to other pentanucleotide repeat mutations (Figure 3A).

In SCA10, the expression of *ATXN10* and its neighboring genes remains unchanged and the pre-mRNA with the expanded AUUCU is spliced normally in affected subjects-derived cells [108]. ATXN10-null mice have shown embryonic lethality, whereas heterozygous mutants have not developed SCA10 phenotype, ruling out a simple loss-of-function of *ATXN10* as the major pathogenic mechanism in SCA10 [108]. The spliced AUUCU expanded RNA repeat forms RNA-protein complexes that aggregate in RNA foci in the cytosol and nuclei of SCA10 human cells and transgenic mouse brains [109,110]. The RNA-binding protein heterogeneous nuclear ribonucleoprotein K (hnRNP K) has been shown co-localizing with nuclear AUUCU foci in SCA10 human cells and transgenic mouse brain. The sequestration of hnRNP K in these RNA foci, likely formed by phase separation [77], changes the splicing regulation of β-tropomyosin and releases protein kinase Cδ that translocates to the mitochondria, leading to apoptosis [109]. Notably, overexpression of hnRNP K in cells expressing expanded AUUCU RNA repeats has been demonstrated to rescue them from apoptosis, showing that the spliced expanded AUUCU RNA repeat triggers neuronal death in SCA10 through loss-of-function of hnRNP K. In interrupted alleles, the inserted pentanucleotide motifs present in affected individuals with epileptic seizures likely recruit additional RBPs relevant for phase separation of condensates with a function in gene regulation of genes associated with epilepsy.

### 2.2. TGGAA Repeat Insertion in BEAN1 and TK2 in SCA31

SCA31, one of the most common types of SCA in Japan, is caused by an insertion of complex pentanucleotide repeats containing (TGGAA)_n_ in an intron belonging to *brain expressed associated with NEDD4-1* (*BEAN1*) and *Thymidine kinase 2* (*TK2*) genes, in chromosome 16q22.1 [34]. The (TGGAA)_n_ in the *BEAN1* strand orientation and the (TTCCA)_n_ in the *TK2* orientation are the only repeats exclusively found in affected individuals among the complex repeat tract seen at this location [34,111].

Of note, the complex repeat is located in the poly-A tail of an AluSx element [34] (Figure 3A). Most of the Japanese subjects have a short (TAAAA)_n_ containing between 8 and 20 repeats (Table 1), whereas sequencing of large insertions in control chromosomes detected a long pure stretch of (TAAAA)_n_ or a complex repeat containing (TAAAA)_n_, (TAGAA)_n_ and (TAAAATAGAA)_n_ [34,86]. SCA31 individuals share the same ancestral haplotype and the 5′-end of pathogenic alleles has a configuration that may vary from TAAAATAGAA(TGGAA)_n_ to TAAAA(TAGAA)_4_(TGGAA)_n_ [112]. This may suggest that pathogenic alleles have arisen by nucleotide A > G [86], or in the opposite strand T > C, substitution similar to what has been proposed for the mutational mechanism of the (ATTTC)_n_ insertion in SCA37 [87].

In SCA31, there is an inverse correlation between the size of the total complex repeat with the (TGGAA)_n_ insertion and the age of disease onset. Furthermore, the total complex repeat with this insertion has a tendency for expansion upon transmission to the youngest generations [34,113]. Apart from Japan, SCA31 is very rare worldwide [86,111,114], but an affected subject has been described in China with the complex (TGGAA)_n_ and the same C > T change in *puratrophin-1* gene found in most Japanese SCA31 individuals [115], showing a strong founder effect.

Clinically, SCA31 is characterized by progressive cerebellar ataxia with a pure cerebellar syndrome [86]. Affected cerebellar tissue on neuropathological examination displayed predominant cerebellar Purkinje cell loss with the remaining Purkinje cells presenting shrinkage of its cell body and amorphous structures with calbindin-positive sprouts surrounding the cell body [86]. RNA foci, likely an aberrant condensate, have been identified in the nuclei of Purkinje cells from brain tissue of affected subjects using probes to target the complex repeat or specifically to the (TGGAA)_n_, in *BEAN1* orientation [34,116]. Expression of the (TGGAA)_n_ in *Drosophila* also causes accumulation of nuclear RNA foci in the fly eye accompanied by eye degeneration [117]. These observations indicate that the (UGGAA)_n_ forms potentially toxic nuclear RNA foci [86].

An in vitro RNA pulldown assay has identified TDP-43, fused in sarcoma (FUS) and hnRNPs as UGGAA-binding proteins, and TDP-43 has further been confirmed to co-localize with nuclear RNA foci in human SCA31 cerebellar Purkinje cells [117]. Co-expression of TDP-43 suppresses fly eye degeneration and decreases the formation of nuclear RNA foci in (UGGAA)_exp_-expressing flies, suggesting that TDP-43 prevents RNA foci formation, resulting in the rescue of (UGGAA)_exp_ RNA toxicity via their direct interactions [117]. Further investigation has demonstrated that the binding of TDP-43 to UGGAA RNA repeat changes its structure and prevents its aggregation, functioning as an RNA chaperone for this RNA repeat. Similar investigations with FUS and hnRNPA2B1 have shown dramatic suppression of eye degeneration and attenuated the formation of RNA foci in (UGGAA)_exp_-expressing flies, confirming their function as RNA chaperones [86,117].

Remarkably the (TGGAA)_n_ is present in Satellite III (SatIII) DNA located in pericentromeric heterochromatin, which is transcribed in a SatIII non-coding RNA. Recent findings suggest that the SCA31 (UGGAA)_n_ RNA competes with long non-coding RNA transcribed from SatIII for SRSF9 splicing factor binding [118], which is abnormally sequestered in SCA31 RNA foci [34]. This SRSF9 is normally recruited to nuclear stress bodies (nSB) with other RBPs, chromatin-remodeling factors, and TFs, resulting in the assembly of nSB, nuclear condensates formed by phase separation, that during thermal stress recovery promote the rapid adaptation of gene expression by target intron retention [119]. 

The small molecule NCD inhibits the assembly of RNA foci, namely releasing TDP-43 from the foci [118]. Although this NCD molecule interferes with the normal assembly of nSB with SRSF9, it is able to suppress eye degeneration in a SCA31 *Drosophila* model, being a therapeutic candidate for the disease [118].

Interestingly, in the *Drosophila* model and in human cerebellar tissues, pentapeptide repeat proteins, originated from the (UGGAA)_exp_, have likely been produced by repeat-associated RAN translation (Figure 2) [117]. These pentapeptide repeat proteins have been detected in the eye imaginal discs of flies and in the cell bodies and dendrites of SCA31 cerebellar Purkinje cells. However, additional investigations are required to understand the role of these pentapeptides in SCA31.

### 2.3. DAB1 ATTTC Repeat Insertion in SCA37

In 2017, we found a pentanucleotide ATTTC repeat insertion in a polymorphic ATTTT repeat as the cause of SCA37 [35]. This ATTTC repeat is in intron 1 or 3 of the 5′ UTR of the *DAB1, reelin adaptor protein* (DAB1) gene, depending on promoter usage [35]. In the reference genome, there is only an (ATTTT)_n_ at this position. Non-pathogenic alleles are very polymorphic, with sizes varying from 7 to 400 ATTTTs (Table 1). On the other hand, in affected subjects, pathogenic alleles have the (ATTTT)_60–79_(ATTTC)_31–75_(ATTTT)_58–90_ configuration (Figure 3B) [120]. The disease is characterized by pure cerebellar ataxia and distinctively onset of dysarthria in late adolescence to adulthood [35,121]. Fifty percent of this variability in age of onset is explained by the size of the (ATTTC)_n_ that shows an inverse correlation with age of disease onset [35]. Both ATTTT and ATTTC repeats are very unstable upon transmission to the next generation. For the (ATTTC)_n_, this intergenerational instability is biased towards expansion as no contraction has been reported [35,122].

In the *DAB1* antisense strand, the (ATTTC)_n_ is in the middle A-rich region of an AluJb element (Figure 3A), which may explain the high instability of this repeat region. Like in other repeat diseases [123,124,125], a small fraction of non-pathogenic repeat alleles are interrupted by other nucleotide motifs, while no interruptions were seen in pathogenic alleles. Interruptions of the (ATTTT)_n_ are AT-rich, with a single A or A(T)_n_, varying from a di- to an octanucleotide interruption [87]. In non-pathogenic alleles, the existence of one shared haplotype for short pure, interrupted, and large pure alleles shows that they may become unstable at both repeat size and sequence [87].

There is a haplotype shared by all Portuguese subjects with SCA37 [35]. This haplotype has been found in two healthy individuals carrying one non-pathogenic (ATTTT)_200_ allele, which, together with the observed instability of normal alleles, suggest that the ATTTC repeat has arisen, first, by lengthening of the ATTTT repeat, followed by one or more nucleotide T > C substitutions, in the last T of the pentanucleotide repeat motif [87]. This mutational mechanism is plausible also for the other ATTTC repeat diseases recently identified [36,37,38,39].

Like in other repeat expansions located in non-coding gene regions, the repeat insertion RNA in SCA37 forms nuclear RNA aggregates in human cells [35]. This has been shown by overexpression of the transfected ATTTC repeat in a human cell line followed by fluorescent in situ hybridization (FISH) with a probe predicted to hybridize to the (AUUUC)_n_ RNA, which detected the widespread formation of nuclear RNA aggregates, 48 h after transfection, not present in cells transfected with the corresponding normal (ATTTT)_7_ or (ATTTT)_139_ alleles. These RNA aggregates are similar to RNA foci, but larger, probably sequester RBPs, forming these aberrant condensates that compete with nuclear membraneless condensates for RBP-binding, disrupting their normal function as happens in SCA31, DM1, DM2, SCA8, and other repeat diseases [1,5,7,75,126].

In vivo, the AUUUC-containing RNA is toxic and impairs early embryonic development in zebrafish embryos [35]. Zebrafish embryos injected with the (AUUUC)_58_ pathological insertion have presented a significantly higher lethality rate compared with embryos injected with control RNA. Strikingly, the number of embryos that developed normally following (AUUUC)_58_ injection has also been significantly lower, demonstrating the high toxicity of this repeat [35]. Sequestration of RBPs by the (AUUUC)_n_ could affect the splicing of several genes crucial for zebrafish development.

*DAB1* encodes an adaptor protein of the reelin signaling pathway that controls accurate positioning of neurons and maturation of synaptic connections in the brain during development [127]. Neuropathological findings in two Spanish subjects with SCA37 have shown severe loss of cerebellar Purkinje cells with abundant astrogliosis [122]. Immunostaining with anti-DAB1 suggested specific overexpression in the SCA37 cerebellum compared with age-matched controls. In control cerebellar tissue, DAB1 was present in soma and dendrites, while SCA37 tissue displayed perisomatic and perinuclear punctate staining in the remaining cerebellar Purkinje cells [122]. Moreover, some Purkinje cells presented abnormal arborization and mispositioning within the granular layer, suggesting that the normal function of the *DAB1* gene is disrupted in SCA37.

## 3. Biallelic *RFC1* AAGGG Expansions in CANVAS

Cerebellar ataxia with neuropathy and vestibular areflexia (CANVAS) is a late-onset slowly progressive recessive disorder characterized by imbalance, sensory neuropathy, bilateral vestibulopathy, and chronic cough [128]. Recently, a biallelic AAGGG repeat expansion in intron 2 of the *Replication factor C subunit 1* (*RFC1*) gene has been identified as the cause of this disease [32,33], estimated to have arisen in Europe more than 25,000 years ago [33].

In the reference genome, there is an (AAAAG)_11_ allele, whereas in affected individuals the expansion size of the novel (AAGGG)_exp_ ranges from 400 to 2,000 repeats (Table 1). In control chromosomes, 75% of the alleles are (AAAAG)_11_, whereas 13% are very polymorphic with large (AAAAG)_n_ sizes that can reach the expansion range; a small fraction (0.8%), however, have (AAGGG)_n_ in the expansion range. The size of the repeat is much larger in (AAGGG)_exp_ alleles, which can reach more than 1,000 units than in (AAAGG)_n_ alleles in the expansion range, that are below 1,000 repeats; most of the (AAAAG)_n_ large alleles have less than 200 units [32]. Three additional repeat structures, AGAGG, AAGAG, and ACAGG have been observed in adult-onset ataxia cases [129,130], as well as a new allele with an (AAAGG)_10-25_ tract preceding the (AAGGG)_n_ [131] further showing the unstable nature of this repeat, being advisable to give careful attention to its sequence and size before releasing a molecular diagnosis.

Of note, in the *RFC1* antisense strand, the novel pentanucleotide repeat is in the poly-A tail of an AluSx3 element (Figure 3A) [32,33]. The role of this Alu element in the high repeat instability seen in *RFC1* alleles is unknown. The large (AAAGG)_n_ alleles in the expansion range often show interruptions and nucleotide changes in the sequence, while 3% of alleles in healthy individuals could not be amplified by standard PCR and have given negative for RP-PCR targeting AAAAG, AAAGG, or AAGGG repeats [32], suggesting the presence of other repeat motifs. In fact, this high instability resembles that observed for other repeat expansions in poly-A regions of Alu elements such as SCA10, DM2, and SCA37 [87,103,107,132].

The disease-associated haplotype has been observed in carriers of two large (AAAGG)_n_ alleles and of (AAGGG)_exp/large_ (AAAGG)_n_ chromosomes [32]. This seems to indicate that the pathogenic (AAGGG)_exp_ allele has arisen by nucleotide A > G substitution, or T > C in the complementary strand, in a large (AAAGG)_n_ chromosome, resembling the mutational mechanism in the origin of SCA37 chromosomes [87] and possibly SCA31, based on analysis of the published data [86,112].

Given the pattern of inheritance and the finding of biallelic expansions, a recessive mode of transmission is likely for CANVAS [32,33]. In Friedrich ataxia, a recessive disease caused by biallelic (GAA)_n_ expansions in the *FXN* gene, there is a decrease in mRNA expression in brain tissue from affected subjects [24,133]. In CANVAS, contrary to what would be expected in a loss-of-function scenario, *RFC1* mRNA assessment in lymphoblasts, muscle, and cerebellar vermis has not shown any significant decrease and no RFC1 protein reduction was detected [32]. Moreover, no formation of abnormal RNA foci in affected brain tissue has been reported [32]. Thus, the mechanism by which such a large pentanucleotide repeat in *RFC1* causes CANVAS remains intriguing.

## 4. Pentanucleotide Repeats in Familial Adult Myoclonic Epilepsy

Familial adult myoclonic epilepsy (FAME), also named familial cortical myoclonic tremor and epilepsy (FCMTE), benign adult familial myoclonic epilepsy (BAFME), and several other designations, has first been described in Japan [134]. The disease is characterized by non-progressive adult-onset of cortical myoclonus and generalized seizures inherited in an autosomal-dominant mode [135]. Usually, myoclonus is the first symptom that manifests between the age of 10 and 60 years by tremulous finger movements and myoclonus of the extremities aggravated by action and posture. Additional manifestations include cerebellar ataxia, dysarthria, cognitive decline, and migraine [134,136]. The diagnosis of FAME is supported by electroencephalographic changes. Interestingly, pathology [137], imaging [138] and clinical presentation suggest also the existence of cerebellar dysfunction [134,139]. There are six known genetic types of FAME and they are all caused by (ATTTC)_n_ insertions [140].

### 4.1. SAMD12 Repeat Insertion in FAME1

In Japanese families with FAME that mapped to the previously known FAME1 locus, on chromosome 8q24 [141,142], an unstable ATTTC repeat with adjacent or flanked ATTTT repeat tracts of variable lengths has been identified in intron 4 of the *Sterile α-motif domain-containing 12* (*SAMD12*) gene [36]. In the reference genome, in the position of this mutation there is ATTTT, but not ATTTC repeats. A total of 82 affected subjects from 48 families have initially shown the ATTTC repeat with adjacent ATTTTs, whereas in 3 affected individuals from one pedigree the ATTTT repeat is flanking the ATTTCs such as in SCA37 (Figure 3B). In fact, long-read sequencing with the Oxford Nanopore Technology identified each of the configurations (ATTTT)_598_(ATTTC)_458_ or (ATTTT)_221_(ATTTC)_225_(ATTTT)_81_ in two affected individuals (Figure 3B). In control subjects, most of the alleles have about 20 ATTTT repeats, while a small fraction has allele sizes larger than 100 repeats (Table 1) [36]. Like in SCA37, no ATTTC repeats have been detected in control subjects [36,143]. Of note, the total length of ATTTT and ATTTC repeat tracts together, determined by Southern blot analysis, correlates with age of onset of epilepsy and age of onset of myoclonic tremor [36].

Interestingly, the repeat is located in the poly-A tail of an AluSq2 element in the *SAMD12* antisense strand (Figure 3A), which may not only contribute to its high instability but be relevant for the FAME1 pathogenesis. In fact, Southern blot analysis of DNA from affected individual autopsy tissues, including brain, liver, and kidney, showed a broad smear, indicative of high somatic instability [36]. Moreover, intergenerational instability has been detected in DNA from peripheral blood leukocytes by Southern blot analysis. The identification by RNA-seq analysis of short reads filled with the repeat motifs ACUUC or GAAGU and AGUUC or GAACU only in the brain and not in liver or LCL of affected subjects, not present in control brain or LCL, is intriguing [36]. The high instability of this repeat is further demonstrated by the recent finding of a Chinese family with a different nucleotide change in the pentanucleotide repeat, a G instead of a C, originating an (ATTTG)_n_ insertion in *SAMD12* [144]. This also confirms that repeat-primed PCR alone cannot guarantee the detection of pentanucleotide repeat insertions [87,120].

The levels of *SAMD12* transcripts in autopsied brains are similar to that of control subjects, but SAMD12 protein is significantly reduced. Nevertheless, transcription of the ATTTC repeat originates an RNA that forms RNA foci in neurons. In autopsy brain tissue, FISH using a probe predicted to hybridize with the AUUUC repeat has allowed the detection of nuclear RNA foci in cortical neurons and in Purkinje cells from affected subjects, but not in neurons from control brains [36]. These nuclear RNA foci in FAME1 brain tissue and the formation of nuclear (AUUUC)_n_ RNA aggregates in human embryonic transfected cells with in vivo toxicity [35] show that the (AUUUC)_n_ likely recruits RBPs originating aberrant nuclear condensates that impair the formation of nuclear membraneless organelles crucial for neurons.

Notably, haplotype analysis has detected a shared disease haplotype among Japanese affected subjects with the two known repeat sequence configurations, suggesting a common ancestral founder. Chinese families with FAME1, presenting the configuration of adjacent ATTTT repeats, have also exhibited a shared core haplotype with Japanese pedigrees [143]. The ATTTC repeat insertion in *SAMD12* has been identified in 50 Japanese and 23 Chinese families and, more recently, two pedigrees of Sri Lankan and Indian origin, and one Thai family have been reported [143,145,146,147,148,149]. All the affected Asian subjects share a core ancestral haplotype and mutation dating based on the length of this haplotype has estimated that it has arisen 12,000–17,000 years ago [148,149]. The old age of this mutation could account for a larger spread than the currently reported, being important to carry out genetic screens for more than one repeat motif. The high instability observed is likely related to the pathogenic RNA that disturbs the physiological function of RBPs recruited for aberrant condensates.

### 4.2. STARD7 ATTTC Repeat Insertion in FAME2

The second locus for FAME has been mapped to chromosome 2p11 in an Italian family [150]. Many previous efforts to identify the gene mutation had been unsuccessful, but the finding of additional pedigrees has allowed to narrow down the candidate region [151,152]. Following the finding of three genes harboring ATTTC repeat insertions causing FAME [36], the effort made to identify the FAME2 gene mutation by analysis of whole-genome sequencing data with ExpansionHunter [153] and exSTRa [154] repeat expansion detection methods has finally been successful. This strategy culminated in the finding of an ATTTC repeat insertion in the first intron of the *Start domain-containing protein 7* (*STARD7*) gene [37]. Oxford Nanopore Technology sequencing of the DNA from two affected individuals identified the (ATTTT)_390_(ATTTC)_345_ and (ATTTT)_340_(ATTTC)_588_ configurations (Figure 3B). The ATTTC repeat insertion has been identified in a total of 22 families with 266 affected individuals. The families are from different origins, but almost all have European ancestry.

The *STARD7* gene encodes a protein involved in lipid transport and metabolism broadly expressed throughout the brain, LCL and fibroblasts. Analysis of the expression levels of *STARD7* transcript and protein assessed by quantitative RT-PCR and Western blot, respectively, in affected subject-derived fibroblasts have shown, however, no significant differences compared with controls [37]. Thus, an RNA gain-of-function, such as in SCA37 and FAME1 [35,36], is the most plausible mechanism for this disease.

### 4.3. MARCH6 Repeat Insertion in FAME3

In 2010, Depienne and colleagues mapped the third locus of FAME to chromosome 5p15, in a large French family [135]. Genomic and RNA expression analysis failed to detect pathogenic variants. Later, the identification of (ATTTC)_n_ insertions causing FAME [36] prompt the search of these repeats within the candidate region, in whole-genome sequencing reads, using ExpansionHunter [153] and other similar bioinformatics tools such as exSTRa [154], STRetch [155] and TRhist [156] programs. This investigation has shown an (ATTTC)_n_ in intron 1 of the *Membrane-associated ring-ch finger protein* (*MARCH6*) gene segregating with the disease in affected individuals with FAME3 [38]. Long-read Oxford Nanopore sequencing has allowed the identification of (ATTTT)_exp_(ATTTC)_exp_ tracts (Figure 3) with between 791 and 1035 repeats, in total (Table 1) [38]. The repeat insertion has been identified in five families of diverse European origins, including French, Dutch, and German.

*MARCH6* encodes an E3 ubiquitin ligase ubiquitously expressed that mediates degradation of misfolded proteins in the endoplasmic reticulum. The (ATTTC)_n_ insertion in *MARCH6* causes no significant changes in gene expression in LCL or fibroblasts of affected individuals when compared with controls [38].

The size of the (ATTTC)_n_ in *MARCH6* inversely correlates with the age of seizures onset. Moreover, the individuals with the largest expansions present the most severe symptoms. Remarkably, two of these individuals have shown micro-rearrangements of the (ATTTC)_n_ and adjacent ATTTTs, resulting in variable repeat configurations [38]. These rearrangements seem to indicate that this large repeat breaks during replication, transcription, or both, but their role in the disease process is still unknown.

### 4.4. Repeat Insertions in Other Types of FAME

The gene for FAME4 has been mapped to chromosome 3q in a large Thai pedigree [157]. More recently, the same methodology of whole-genome long-read sequencing followed by searching repeat motifs in the sequencing data allowed the finding of an ATTTC repeat with adjacent ATTTTs in intron 1 of *Yeats domain-containing protein 2* (*YEATS2*) gene causing FAME4 [39]. At this genomic location, most control subjects have from 4 to 119 ATTTT repeats, while very few have approximately 1000 ATTTTs (Table 1). In an affected subject, the complete sequencing of the pathogenic allele has shown the configuration (ATTTT)_819_(ATTTC)_221_ (Figure 3B).

In Japanese FAME families without the ATTTC insertion in *SAMD12* the search for ATTTC repeats in ATTTT repeat regions of the reference genome has allowed FAME6 gene identification. Expansion of an ATTTC repeat in a 5′ UTR intron of *Trinucleotide repeat-containing 6A* (*TNRC6A*) gene, on chromosome 16p, has been identified in one family by searching whole-genome sequencing data followed by Southern blot analysis [36]. In the reference genome, there is only an (ATTTT)_18_ allele in this repeat location. The ATTTC repeat is flanked by ATTTT repeat tracts (Figure 3B) originating the configuration (ATTTT)_22_(ATTTC)_exp_(ATTTT)_exp_. In control subjects, there are no ATTTC repeat tracts (Table 1).

The strategy used to identify the ATTTC repeat insertion in FAME6 has successfully enabled the finding of another ATTTC repeat insertion in intron 14 of *Rap guanine nucleotide-exchange factor 2* (*RAPGEF2*) gene, on chromosome 4q32.1, in a Japanese family with FAME7 [36]. Again, in the reference genome, there is only an ATTTT repeat in this position. In affected subjects, the ATTTC is also flanked by ATTTT repeats (Figure 3B). The control subjects analyzed have shown no ATTTC repeats. This disease has also been identified in a Chinese family, showing the same structure of (ATTTC)_n_ flanked by ATTTT repeats [145].

## 5. Alu Repeat Expansions and Insertions

Analysis of pentanucleotide repeat flanking regions shows that they are all, except in FAME7 *RAPGEF2*, located in Alu elements (Figure 3A) [34,35,36,37,38,39,87,107]. Alu elements are primate-specific retrotransposons that successfully colonized these genomes by the accumulation of new insertions. These elements, with approximately 300pb, compose about 11% of the human genome [158]. They are composed of two monomers and two poly-As, one in the middle and the other in the 3′-end of the element (Figure 3A). Alu elements are divided into three subfamilies according to their age, AluJ is the oldest, AluS, the intermediate, and AluY, the most recent [159]. The oldest Alu elements accumulated mutations in their sequence, including in their poly-As, being previously associated with the birth and expansion of microsatellite repeats [132,160,161]. These elements were more active in retrotransposition during earlier primate evolution, but they continue to be transcribed and inserted into human genomes with an impact on biology and pathology [159]. Besides the consequences of their retrotransposition, Alu RNAs not only can regulate gene expression [162], but they can drive nuclear localization and fate of transcripts, including long non-coding RNAs and mRNAs, with integrated Alu elements [163]. This has further been shown to be achieved by interaction with an RBP, hnRNP K [163]. The mechanism by which hnRNP K affects nuclear enrichment is not known, but the recent discoveries [71,72] allow us to propose that Alu-containing RNAs could have a say in the formation of nuclear condensates. In Alu orientation, the Alu pentanucleotide repeat motifs differ only by one nucleotide in all pathogenic repeats except CANVAS *RFC1*, which suggests that they could drive neurodegeneration by a similar mechanism of RNA gain-of-function and likely by disruption of the function of the Alu element, compromising neuronal gene expression of specific genes, a hypothesis that needs to be tested.

## 6. Therapeutic Strategies

One of the most promising therapeutic strategies for diseases caused by transcribed repeat expansion relies on antisense oligonucleotides (ASOs) [164,165,166,167]. ASOs are short nucleotide sequences chemically modified that bind to the target RNA repeat by Watson–Crick base-pairing, modulating splicing, inhibiting translation, preventing RBP aggregation, or promoting RNA degradation mediated by RNase-H [164,168]. ASOs delivery to the nervous system as well as their stability has led to two critical challenges for therapeutic intervention. Therefore, several delivery carriers and chemical modifications have been studied to increase ASOs uptake and nuclease-resistance [164,167,169,170].

ASOs have shown encouraging results for several neurodegenerative and neuromuscular disorders [171,172,173,174,175,176,177,178,179,180,181,182,183]. In preclinical studies, non-allele specific ASOs acting by RNase-H degradation have been successful for SCA1 [171], SCA3 [173,174], SCA7 [177] and DM1 [178]. In addition, ASOs leading to polyQ exon skipping [175,176], namely combining two ASOs for *ATXN3* exon 9 and 10 skipping, have shown therapeutic value in SCA3 [176].

The most promising ASOs reached clinical trials phase I, being the case of ASOs BIIB105 (NCT04494256) for ALS, and both WVE-004 (NCT04931862) and BIIB078 (NCT03626012) for *C9ORF72* FTD/ALS [172,179,180]. Interestingly, the ASO BIIB105 has been designed against polyQ-*ATXN2* for the treatment of ALS subjects carrying an *ATXN2* intermediate allele (30-33 CAG/CAA repeats) [172]. Although several nonspecific-allele ASOs targeting both pathogenic and non-pathogenic RNA repeats have been successful with minor effects in wild-type transcript function [167], ASOs targeting single nucleotide polymorphisms in linkage disequilibrium with expanded alleles have also been tested in preclinical studies for SCA3 [181] and in clinical trials for HD (WVE-003, NCT05032196) [182], lowering the levels of mutant RNAs.

To this date, no studies have been reported with ASOs for the treatment of pentanucleotide repeat diseases. Instead, small molecule compounds have successfully targeting the SCA10 RNA repeat [183] and SCA31 RNA repeat insertion [118], inhibiting RNA foci formation in vitro. An increasing number of studies have shown that both sense and antisense transcripts are involved in pathogenesis, therefore, the development of ASOs targeting both sense and antisense RNAs may be required for more efficient rescue of disease phenotype [84].

## 7. Conclusions and Future Perspectives

Pathogenic pentanucleotide repeats have been identified in three different types of neurological diseases, SCAs, CANVAS, and FAMEs. These pathogenic repeats are all in non-coding gene regions, thus being transcribed in the same orientation of their host gene, but not translated as part of the gene protein. In the host gene antisense strand, they are located in Alu elements, but these Alu transcripts are difficult to grasp and have not been reported. In SCA31 and FAME1 the pathogenic repeat forms RNA foci in the nuclei of cerebellar Purkinje cells that, in SCA31, interact with the ALS protein TDP-43. In SCA10, the nuclear RNA foci sequester hnRNP K causing its loss of function and leading to apoptosis. The AUUUC RNA repeat forms intranuclear RNA foci in transfected human embryonic cells and causes lethal zebrafish developmental malformations in SCA37, whereas FAME1 triggers the formation of nuclear RNA foci in the nucleus of Purkinje and cortical neurons in affected brain tissue. RNA foci are formed by multivalent intermolecular base-pairing of RNAs with repeated nucleotide motifs that recruit RBPs and phase separate in cellular condensates. As all but one pathogenic pentanucleotide repeat is located in Alu elements, differ by only one nucleotide, and, at least, four of them originate the formation of nuclear RNA foci with RBPs that drive nuclear localization, we hypothesized that each one could be toxic through the formation of aberrant nuclear condensates. Thus, in pentanucleotide SCAs and FAME, the data gathered support a role for the pathogenic RNA in neuronal dysfunction by an RNA-gain-of function mechanism. The specificity of the disease alterations likely results from temporal and spatial differences in cellular and tissue gene expression, namely the levels of expression of the affected gene together with free RBP levels for interaction. Furthermore, the abnormal RAN pentapeptides in cell bodies and dendrites of Purkinje cells from SCA31 cerebellar tissue suggest that this mechanism deserves further investigation in the other pentanucleotide repeat disorders. Moreover, since these pentanucleotide repeat diseases present some clinical, genetic, or neuropathological overlap, shared mechanisms underlying the neuronal pathology are expected. The new opportunities for research created by the discovery of pathogenic pentanucleotide repeats will advance knowledge regarding the complexity of mysterious non-coding gene regions in the human genome and their essential role in neurons.

## Figures and Tables

**Table 1:** Neurological diseases caused by pentanucleotide repeat expansions.

Disease	Type	Gene	Pathogenic Alleles	Non-Pathogenic Alleles	
				Common	Less Common
SCA	^27^SCA10	*ATXN10*	(ATTCT)_800–4500_	(ATTCT)_10–16_	(ATTCT)_17–32_
	^34^SCA31	*BEAN1*	TAAAA(TAGAA)_1–4_(TGGAA)_n_	(TAAAA)_8–20_	(TAAAA)_exp_
					(TAAAA)_n_(TAGAA)_n_ and (TAAAATAGAA)_n_
	^35^SCA37	*DAB1*	(ATTTT)_60–79_(ATTTC)_31–75_(ATTTT)_58–90_	(ATTTT)_7–30_	(ATTTT)_31–400_
CANVAS	^32^CANVAS	*RFC1*	(AAGGG)_400-2000_	(AAAAG)_11_	(AAAAG)_15–200_
					(AAAGG)_40–1000_
			^130^(ACAGG)_~1000_		
			^131^(AAAGG)_10–25_(AAGGG)_exp_		
FAME	^36^FAME1	*SAMD12*	(ATTTT)_exp_(ATTTC)_exp_	(ATTTT)_<100_	(ATTTT)_>100_
			[(ATTTT)_exp_(ATTTC)_exp_(ATTTT)_exp_]_440–3680_		
			^143^(ATTTT)_n_(ATTTG)_n_		
	^37^FAME2	*STARD7*	(ATTTT)_340–390_(ATTTC)_345–588_	(ATTTT)_n_	n.d.
	^38^FAME3	*MARCH6*	[(ATTTT)_exp_(ATTTC)_exp_]_791–1035_	(ATTTT)_9–20_	n.d.
	^39^FAME4	*YEATS2*	(ATTTT)_819_(ATTTC)_221_	(ATTTT)_4–120_	(ATTTT)_121–1219_
	^36^FAME6	*TNRC6A*	(ATTTT)_22_(ATTTC)_exp_(ATTTT)_exp_	(ATTTT)_<200 bp *_	(ATTTT)_>200 bp *_
	^36^FAME7	*RAPGEF2*	(ATTTT)_exp_(ATTTC)_exp_(ATTTT)_n_	(ATTTT)_<300 bp *_	(ATTTT)_300–3000 bp *_

n.d.—not determined; * Repeat size is in base pairs.

## Data Availability

Not applicable.
